# Expressions of P-Glycoprotein, Multidrug Resistance Protein 1 and Annexin A2 as Predictive Factors for Intravesical Recurrence of Bladder Cancer after the Initial Transurethral Resection and Immediate Single Intravesical Instillation of Adriamycin

**DOI:** 10.31557/APJCP.2021.22.5.1459

**Published:** 2021-05

**Authors:** Harutake Sawazaki, Keiichi Ito, Takako Asano, Kenji Kuroda, Akio Horiguchi, Hitoshi Tsuda, Tomohiko Asano

**Affiliations:** 1 *Department of Urology, National Defense Medical College Tokorozawa, Japan. *; 2 *Department of Basic Pathology, National Defense Medical College Tokorozawa, Japan. *

**Keywords:** Bladder cancer, intravesical recurrence, Adriamycin, P-glycoprotein

## Abstract

**Objective:**

Immediate single instillation of chemotherapy following transurethral resection of bladder tumor (TURBT) is suggested for non-muscle invasive bladder cancer (NMIBC) patients. However, no study has evaluated molecular marker that was involved in intravesical recurrence (IVR) after single instillation of chemotherapy. Therefore, this study aimed to evaluate whether P-glycoprotein, multidrug resistance protein 1 (MRP1), Annexin A2 (ANXA2) or nucleophosmin (NPM) expression predicts IVR after initial TURBT and immediate single intravesical adriamycin instillation.

**Methods:**

We retrospectively reviewed consecutive 443 patients who underwent TURBT. Of these, 54 patients who underwent initial TURBT and single instillation of adriamycin for NMIBC were included. The expressions of P-glycoprotein, MRP1, ANXA2 and NPM were evaluated immunohistochemically and were divided into 2 groups (low or high) according to the staining intensity and/or proportion of positive cells. IVR was assessed by Kaplan-Meier method. Cox`s multivaritate analyses were performed to identify independent predictors for IVR.

**Results:**

Nineteen patients (35.1%) had IVR. High P-glycoprotein expression was significantly correlated with multiplicity, pT stage and high grade. High ANXA2 expression was significantly correlated with high grade. MRP1 and NPM were not correlated with any clinicopathological variables. MRP1 expression and ANXA2 expression were significantly correlated with P-glycoprotein expression. Patients with high P-glycoprotein expression had significantly worse IVR-free survival (IVRFS) than those with low P-glycoprotein expression (P =0.015). The difference in IVRFS rates between patients with high ANXA2 expression and those with low ANXA2 expression was nearly significant (P =0.057). Univariate analyses indicated multiplicity, high grade and high P-glycoprotein expression were significant predictors for IVR. Multivariate analysis indicated high grade was an independent predictor for IVR.

**Conclusions:**

High P-glycoprotein expression was associated with IVR. Further study was needed to determine significance of P-glycoprotein expression in IVR after single intravesical adriamycin instillation.

## Introduction

GLOBOCAN data revealed that an estimated 550,000 people were diagnosed with bladder cancer globally in 2018. This accounts for roughly 3% of all new cancer diagnoses. About 70% of bladder cancer is non-muscle invasive bladder cancer (NMIBC) and remaining 30% is muscle invasive bladder cancer (MIBC) (Saginala et al., 2020). For the patients with NMIBC, transurethral resection of bladder tumor (TURBT) with intravesical bacillus Calmette-Guérin (BCG) instillation or prophylactic intravesical instillation chemotherapy is chosen, whereas for the patients with MIBC, radical cystectomy with neoadjuvant chemotherapy is chosen. For the patients with advanced bladder cancer, systemic cisplatin-based chemotherapy is chosen (Kamat et al., 2016).

Adriamycin is commonly used in chemotherapeutic treatment of bladder cancer including systemic chemotherapy (the regimen of methotrexate, vinblastine, adriamycin, cisplatin) and intravesically administered chemotherapy (Mitsui et al., 2012; Nargund et al., 2012). The risk of intravesical recurrence (IVR) was reduced to 0.21 relative to the control group by prophylactic intravesical adriamycin instillation after TURBT (Obata et al., 1994). Tumor cells are considered to have acquired resistance to adriamycin when post-treatment IVR occurs. The phenomenon of multidrug resistance (MDR) to anticancer agents is a major obstacle to overcome, and further investigations on MDR in bladder cancer are required (Meng et al., 2013). 

A lot of candidate molecules that are correlated with or involved in MDR mechanisms have been identified. The appearance of tumor cells resistant to anticancer agents is often associated with increased expression of 2 representative adenosine triphosphate (ATP) binding cassette (ABC) superfamily proteins, P-glycoprotein and multidrug resistance protein 1 (MRP1). mRNA and protein levels of P-glycoprotein and MRP1 were reportedly over-expressed in recurrent tumors after TURBT plus prophylactic intravesical adriamycin instillation and in residual tumors after systemic chemotherapy using methotrexate, vincristine, adriamycin and cisplatin (Tada et al., 2002).

Annexin A2 (ANXA2) belongs to annexin superfamily of Ca2+-dependent phospholipid–binding proteins and is involved in invasion, angiogenesis and migration in cancer cells (Xu et al., 2015). ANXA2 expression in adriamycin-resistant bladder cancer cell line was significantly higher than that in normal bladder epithelium. ANXA2 overexpression in bladder cancer tissues was significantly correlated with invasion depth and shorter recurrence-free survival (Hu et al., 2016).

Nucleophosmin (NPM) is a nucleolar phosphoprotein with pleiotropic functions in various cellular processes, such as ribosome biogenesis, centrosome duplication, cell cycle progression, apoptosis and cell differentiation (Lindstrom, 2011). NPM is higher expressed in cancer cells compared to normal cells. Higher NPM expression was correlated with more advanced pathological stages, higher tumor grades, and poor prognosis and recurrence in bladder cancer tissues. High NPM expression was correlated with poor prognosis and recurrence (Tsui et al., 2008). Overexpressions of ANXA2 and NPM were identified in adriamycin-resistant human bladder cancer cell lines by proteome analysis. Therefore, both ANXA2 and NPM may also take part in the mechanism of MDR in bladder cancer (Meng et al., 2013). 

Immediate single instillation of chemotherapy after TURBT might prevent IVR. Possible mechanisms are the eradication of floating tumor cells, an ablative effect (chemo resection) on residual tumor cells at the resection site, and an ablative effect on small overlooked tumors (Bosschieter et al., 2018). In the EAU (European Association of Urology) and AUA (American Association of Urology) guidelines, single instillation of chemotherapy is suggested for NMIBC patients with low- or intermediate- risk. CUA (Canadian Urological Association) and NICE (National Institute for Health and Care Excellence) guidelines recommends single instillation of chemotherapy in all patients with NMIBC (Zamboni et al., 2018). To our knowledge, no study has evaluated molecular marker that was involved in IVR after immediate single instillation of chemotherapy following TURBT in NMIBC patients. In our institution, TURBT and immediate single intravesical adriamycin instillation have been performed to NMIBC patients. The aims of this study were to investigate whether P-glycoprotein, MDR1, ANXA2 or NPM expression predicts IVR after initial TURBT and single postoperative intravesical adriamycin instillation. 

## Materials and Methods

We reviewed consecutive 443 patients who had undergone TURBT at our institution from January 2008 to June 2014. Among 443 patients, 237 patients had undergone initial TURBT. Of these, 54 patients who had undergone initial TURBT and immediate (within 24 h after TURBT) single postoperative intravesical adriamycin instillation for NMIBC were included in this study. 54 patients included 44 males and 10 females whose ages ranged from 51 to 94 (median age, 71). TURBT was performed according to the standard procedure. To keep the resection quality, the presence of detrusor muscle in the specimen was required. Second TURBT was performed in patients with T1/grade 3 tumors. Cauterisation was avoided as much as possible during TURBT to avoid the tissue deterioration. In our institution, single intravesical adriamycin instillation was performed by two patterns (adriamycin 60 mg in 30 mL of normal saline or adriamycin 80 mg in 1000 mL of normal saline). Adriamycin 60 mg in 30 mL of normal saline was delivered into the bladder through a catheter and was retained for one hour in 20 patients. Adriamycin 80 mg in 1,000 mL of normal saline was delivered into the bladder through a 3-way catheter for 12 hours in 34 patients. None of these patients received additional intravesical chemotherapy or BCG instillation. 

Pathological characteristics were assessed using the 2009 TNM classification and the 1998 World Health Organization grading system. All patients had pure urothelial carcinoma. The histological grade was grade 1 in 8 patients, grade 2 in 32 patients and grade 3 in 14 patients. Pathological T (pT) stage was pTa in 48 patients and pT1 in 6 patients. There was no patient whose tumor was concomitant with carcinoma in situ. The number of tumor was solitary in 37 patients and multiple in 17 patients. Tumor size was less than 3 cm in 44 patients and 3 cm more in 10 patients. According to the EAU guideline, all patients were categorised in low-risk (n=24), intermediate-risk (n=15), and high-risk groups (n=15). 

Follow-up investigations consisted of urinary cytology and cystoscopy. The patients were evaluated every 3 months for the first 2 years and every 6-12 months thereafter. Intravesical recurrence-free survival (IVRFS) was evaluated using the date when IVR was found. This study was approved by the institutional review board (No. 2693).

*Immunohistochemistry*

Immunohistochemistry was performed using 4 µm-thick sections cut from formalin-fixed, paraffin-embedded blocks. Immunohistochemistry was performed as previously described (Hamada et al., 2014). The primary antibodies used were a mouse anti-P-glycoprotein monoclonal antibody (C219, Calbiochem, CA, USA) at 1:50 dilution, a mouse anti-MRP1 monoclonal antibody (MRPm5, KAMIYA Biomedical Company, CA, USA) at 1:100 dilution, a rabbit anti-ANXA2 polyclonal antibody (11256-1-AP, Proteintech, IL, USA) 1:50 dilution, and a mouse anti-NPM monoclonal antibody (FC-61991, Thermo Fisher Science, MA, USA) at 1:100 dilution overnight at 4ºC. The samples incubated without the primary antibody were also stained using the same method and used for baseline staining. Human liver tissue was used as positive control for anti-P-glycoprotein antibody. Human testicular tissue was used as positive control for anti-MRP1 antibody. Human colon cancer tissue was used as positive control for anti-ANXA2 antibody and for anti-NPM antibody.

Two investigators (H.S. and K.I.) blinded to the patients’ clinical course independently evaluated the stained slides. The P-glycoprotein and the MRP1 expression status were scored as positive when more than 10% of tumor cells stained, intermediate when positive staining in 10% or less of tumor cells or diffusely weak staining, or negative when no staining, as previously described (Diestra et al., 2003). The P-glycoprotein and the MRP1 expression status were finally categorized into two groups: low expression when intermediate or negative, and high expression when positive. ANXA2 expression status was categorized into two groups, low and high. Tumors were considered high expression if at least 25% of the tumors demonstrated strong staining for ANXA2, as previously described (Inokuchi et al., 2009). NPM expression was categorized into 3 groups according to the staining intensity. The intensity of staining was classified into three levels: level 1, no or faint nucleus staining; level 2, weak nucleus staining; level 3, strong nucleus staining, as previously described (Sawazaki et al., 2016). The NPM expression status was finally categorized into two groups: low expression when level 1 or 2, and high expression when level 3. 

*Statistical analysis*

Chi-square test was used to compare the immunoreactivity with the clinicopathological features. Kaplan-Meier analysis was performed to calculate IVRFS, and the differences in survival curves were analyzed by the log-rank test. Univariate and multivariate analyses were performed using Cox’s proportional hazard regression model. All tests were carried out with SPSS® version 24 (IBM Corp., Armonk, NY). P < .05 was considered statistically significant.

## Results

The median follow-up was 28 months (range 7-123). Nineteen patients (35.1%) had IVR. There was not significant difference of IVR between patients with bolus adriamycin instillation and patients with continuous adriamycin irrigation (P = 0.493). 

**Figure 1:**
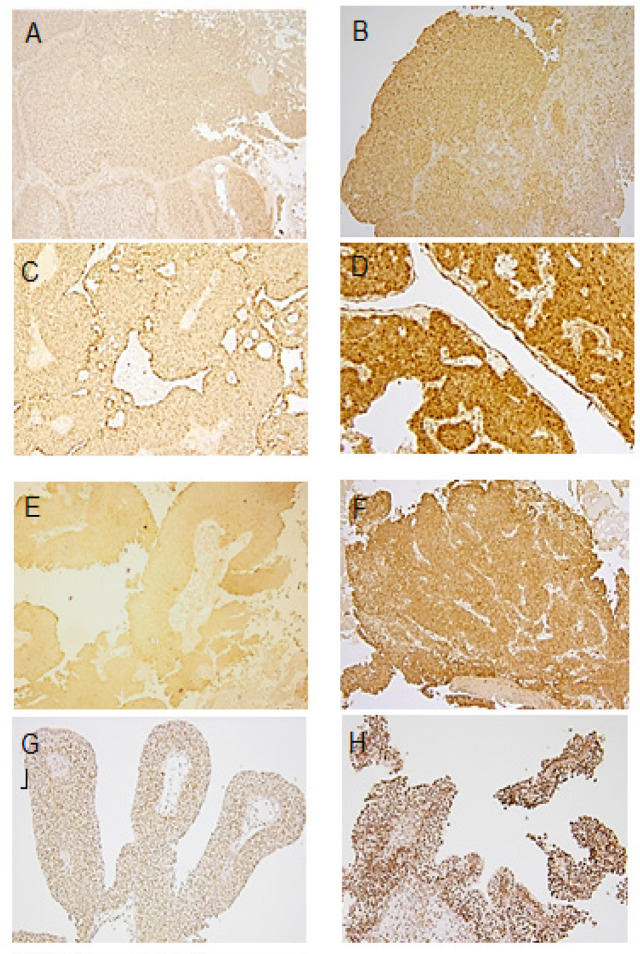
Results of Immunohistochemistry in Paraffin Embedded Bladder Cancer Specimens for P-glycoprotein (A and B), multidrug resistance protein 1 (MRP1) (C and D), annexin A2 (ANXA2) (E and F), and nucleophosmin (NPM) (G and H). (A) Faint cytoplasmic staining for P-glycoprotein. (B) Strong staining of membrane and cytoplasm for P-glycoprotein. (C) Weak cytoplasmic staining for MRP1. (D) Strong cytoplasmic staining for MRP1. (E) Weak cytoplasmic staining for ANXA2. (F) Strong cytolpasmic staining for ANXA2. (G) Weak nuclear staining for NPM. (H) Strong nuclear staining for NPM. Magnification ×100

High P-glycoprotein and high MRP1 expressions were seen in 13 tumors (24.0%) and 17 tumors (31.4%), respectively (Figure 1). High P-glycoprotein expression was significantly correlated with multiplicity (P = 0.019), pT stage (P = 0.001), and high tumor grade (P = 0.002) (Table 1). MRP1 expression was not correlated with any evaluated clinicopathological variables. MRP1 expression was significantly correlated with P-glycoprotein expression (P < 0.001).

High ANXA2 expression and high NPM expression were seen in 19 tumors (35.1%) and 22 tumors (40.7%), respectively (Figure 1). High ANXA2 expression was significantly correlated with high tumor grade (Table 1). NPM expression was not correlated with any evaluated clinicopathological variables. ANXA2 expression was significantly correlated with P-glycoprotein expression (P < 0.001), but NPM expression was not correlated with P-glycoprotein expression (P = 0.910).

**Figure 2:**
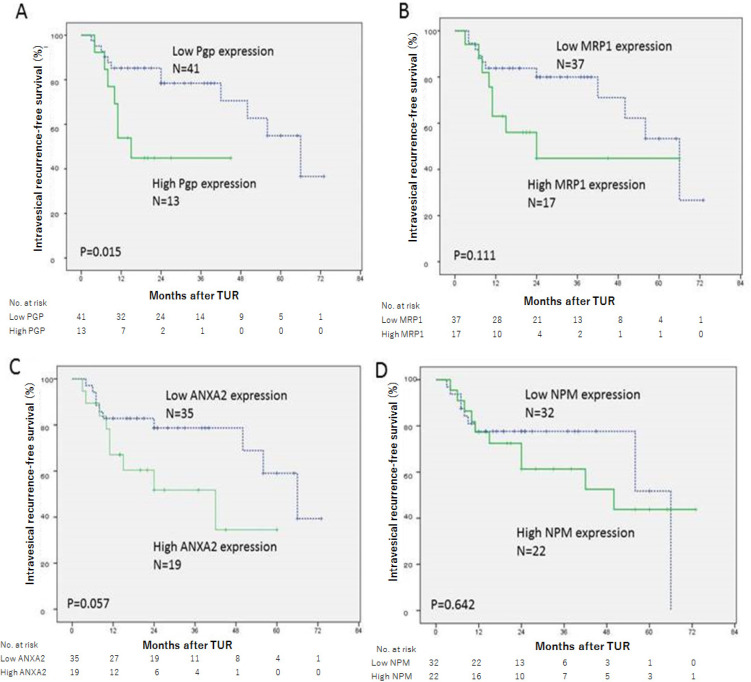
Intravesical Recurrence-Free Survival Curves of the Patients with Bladder Cancer after the Initial TURBT and Immediate Single Intravesial Adriamycin Instillation. (A) Curves stratified by P-glycoprotein expression status. (B) Curves stratified by MRP1 expression status. (C) Curves stratified by ANXA2 expression status. (D) Curves stratified by NPM expression status. Two curves differ significantly between high and low P-glycoprotein expression groups (P = 0.015) in (A). Two curves tend to differ between high and low ANXA2 expression groups but the difference was not significant (P = 0.057) in (C). Two curves did not differ in (B) and (D). Pgp, P-glycoprotein; MRP1, multidrug resistance protein 1; ANXA2, Annexin A2; NPM, nucleophosmin

Patients with high P-glycoprotein expression had significantly worse IVRFS than those with low P-glycoprotein expression (P = 0.015). The 2-year IVRFS rates of high- and low-P-glycoprotein patients were 44.9% and 78.4%, respectively (Figure 2A). There was no significant difference in IVRFS rates between patients with high- and low-MRP1 expression (P = 0.111) (Figure 2B). IVRFS rates tended to be different between patients with high ANXA2 expression and those with low ANXA2 expression although the difference was not statistically significant (P = 0.057). The 2-year IVRFS rates of high ANXA2 and low ANXA2 patient groups were 51.8% and 78.7%, respectively (Figure 2C). There was no significant difference in the IVRFS rates between patient groups with high- and low-NPM expression (P = 0.642) (Figure 2D). 

Cox`s univariate analyses indicated that multiplicity, high tumor grade, and high P-glycoprotein expression were significant predictors for IVR. pT stage, tumor size and high ANXA2 expression were also nearly significant predictors for IVR although the impact was not significant statistically. A multivariate analysis including these three parameters indicated only high tumor grade was an independent predictor for IVR (Table 2).

**Figure 3:**
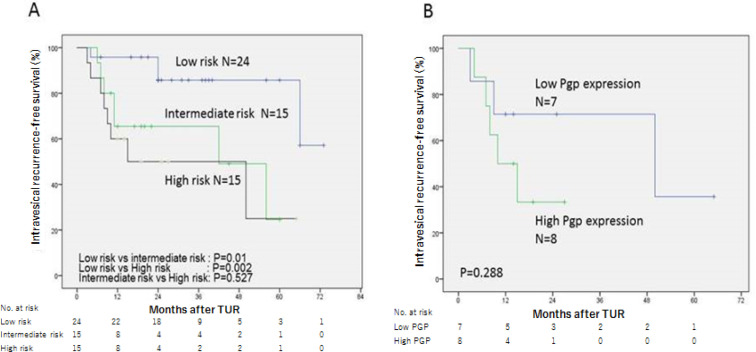
Kaplan-Meier Curves for Intravesical Recurrence-Free Survival of NMIBC Patients after Immediate Single Intravesial Adriamycin Instillation. Curves stratified by risk stratification (B) Curves stratified by P-glycoprotein expression status in high-risk patients. Pgp, P-glycoprotein

According to the EAU guideline, all patients (n=54) were categorised in low-risk (n=24), intermediate-risk (n=15), and high-risk groups (n=15). The 2-year IVRFS rates were 85.7% in low-risk, 65.5% in intermediate-risk, and 50.0% in high-risk groups, respectively (Figure 3A). The percentages of patients with high P-glycoprotein expression were 0% (0/24) in low-risk, 33.3% (5/15) in intermediate-risk, and 53.3% (8/15) in high-risk, respectively. In the intermediate-risk group, there was no significant difference in the IVRFS rates between patient groups with high- and low-P-glycoprotein expression (P = 0.839). In the high-risk group, IVRFS rate of patients with high P-glycoprotein expression tended to be lower than that of patients with low P-glycoprotein expression (P = 0.288) (Figure 3B).

**Table 1:** Relationship between Expression of Four Molecular Markers and Clinicopathological Characteristics of Patients with Bladder Cancer Treated with TURBT and Immediate Single Intravesical Adriamycin Instillation

	No. of patients (%)								
Parameter	Total	High Pgp expression	P	High MRP1 expression	P	High ANXA2 expression	P	High NPM expression	P
	54	13 (24.0)		17 (31.4)		19 (35.1)		22 (40.7)	
Age (years)			0.61		0.74		0.75		0.21
<70	24 (44.4)	5 (9.2)		7 (12.9)		9 (16.6)		12 (22.2)	
≥70	30 (55.5)	8 (14.8)		10 (18.5)		10 (18.5)		10 (18.5)	
Gender			0.93		0.79		0.47		0.76
Male	44 (81.4)	11 (20.3)		14 (25.9)		14 (25.9)		18 (33.3)	
Female	10 (18.5)	2 (3.7)		3 (5.5)		5 (9.2)		4 (7.4)	
Smoking history			0.74		0.83		0.96		0.59
Yes	32 (59.2)	7 (12.9)		9 (16.6)		11 (20.3)		12 (22.2)	
No	20 (37.0)	6 (11.1)		7 (12.9)		7 (12.9)		9 (16.6)	
Unknown	2 (3.7)	0 (0)		1 (1.8)		1 (1.8)		1 (1.8)	
Multiplicity			0.019*		0.46		0.06		0.25
Solitary	37 (68.5)	5 (9.2)		10 (18.5)		10 (18.5)		17 (31.4)	
Multiple	17 (31.4)	8 (14.8)		7 (12.9)		9 (16.6)		5 (9.2)	
Tumor size (cm)			0.37		0.79		0.47		0.68
<3	44 (81.4)	9 (16.6)		13 (24.0)		14 (25.9)		19 (35.1)	
≥3	10 (18.5)	4 (7.4)		4 (7.4)		5 (9.2)		3 (5.5)	
pT			0.001*		0.56		0.72		0.96
pTa	48 (88.8)	8 (14.8)		14 (25.9)		16 (29.6)		20 (37.0)	
pT1	6 (11.1)	5 (9.2)		3 (5.5)		3 (5.5)		2 (3.7)	
Grade			0.002*		0.16		0.02*		0.61
low	40 (74.0)	5 (9.2)		10 (18.5)		10 (18.5)		15 (27.7)	
high	14 (25.9)	8 (14.8)		7 (12.9)		9 (16.6)		7 (12.9)	

Pgp, P-glycoprotein; MRP1, multidrug resistance protein 1; ANXA2, Annexin A2; NPM, nucleophosmin; *, Statistically significant

**Table 2:** Univariate and Multivariate Cox`s Proportional Hazard Regression Analysis Results for Intravesical Recurrence-Free Survival

		Univariate			Multivariate		
Parameter		P	HR	95% CI	P	HR	95% CI
Age, y	(70≥ vs 70<)	0.426	0.689	0.275-1.724			
Gender	(male vs female)	0.308	0.584	0.208-1.642			
Smoking history	(positive vs negative)	0.646	0.894	0.553-1.444			
Tumor size	(≥3cm vs <3cm)	0.087	2.509	0.876-7.188	0.309	1.905	0.551-6.587
Multiplicity	(multiple vs solitary)	0.030*	2.810	1.106-7.143	0.058	2.931	0.965-8.901
pT stage	(pT1 vs pTa)	0.076	3.255	0.885-11.964	0.913	0.899	0.133-6.058
Grade	(high vs low)	0.004*	3.935	1.549-9.996	0.026*	3.484	1.159-10.474
Pgp expression	(high vs low)	0.023*	3.245	1.179-8.930	0.875	1.269	0.064-4.25.08
MRP1 expression	(high vs low)	0.121	2.082	0.824-5.260	0.288	2.006	0.556-7.245
ANXA2 expression	(high vs low)	0.066	2.416	0.942-6.196	0.639	0.526	0.036-7.727
NPM expression	(high vs low)	0.645	1.240	0.496-3.098			

Pgp, P-glycoprotein; MRP1, multidrug resistance protein 1; ANXA2, Annexin A2; NPM, nucleophosmin; *, Statistically significant

## Discussion

In the present study, we evaluated the association between expression of the four proteins and IVR in patients with initial TURBT and immediate single postoperative intravesical adriamycin instillation. High P-glycoprotein expression was significantly correlated with high tumor grade. Patients with high P-glycoprotein expression had significantly higher IVR rate than patients with low P-glycoprotein expression. A univariate analysis indicated that high P-glycoprotein expression was a significant predictive factor for IVR, but the multivariate analysis indicated only high tumor grade was an independent predictor for IVR. High ANXA2 expression was nearly associated with IVR and was also significantly correlated with high P-glycoprotein expression.

P-glycoprotein is a member of the ATP-dependent membrane transport proteins (ATP-Binding Cassette transporters) and is known to pump substrates out of cells in ATP-dependent mechanism. The overexpression of P-glycoprotein in tumor cells reduces the intracellular drug concentrations, which decreases the cytotoxicity of a broad spectrum of antitumor drugs (Abdallah et al., 2015). P-glycoprotein, MRP1 and MRP3 mRNA expressions in clinical samples of bladder cancer were significantly correlated with drug resistance of the cancer cells to adriamycin. In particular, P-glycoprotein mRNA expression had the strongest correlation with resistance to adriamycin (Tada et al., 2002). In that study, P-glycoprotein mRNA level was well correlated with its protein expression level using immunohistochemical analysis. In the present study, high P-glycoprotein expression was significantly associated with IVR. P-glycoprotein mRNA levels were significantly higher in high-grade (G3) tumors than in well to moderate (G1-2) tumors in untreated stage, Ta-T4 bladder cancer samples (Clifford et al., 1994). There was no significant correlation between P-glycoprotein mRNA levels and prognosis or recurrence, but a proportion of survivors for patients with high P-glycoprotein mRNA tumors was lower than that with low P-glycoprotein mRNA tumors (p = 0.07). In our study, high P-glycoprotein expression was significantly correlated with high tumor grade (G3) and was observed in 57.0% of G3 bladder tumors. P-glycoprotein expression protein levels in initial NMIBC were associated with IVR after immediate single instillation of adriamycin.

MRP1 also belongs to the superfamily of ATP-Binding Cassette transporters and helps the cells to survive by pumping out toxic compounds (Borst et al., 1999). Induction of P-glycoprotein gene expression is more important than that of MRP1 gene expression with respect to resistance to adriamycin in clinical samples of bladder cancer (Tada et al., 2002). In our study, MRP1 expression was not significantly associated with IVR.

ANXA2 is a calcium-dependent phospholipid binding protein and is expressed in a wide spectrum of cancers, and exerts profound effects on tumor cell adhesion, proliferation, apoptosis, invasion and metastasis as well as tumor neovascularization (Xu et al., 2015). ANXA2 overexpression was seen in an adriamycin-resistant bladder cancer cell line. In clinical samples of bladder cancer, ANXA2 expression in T2 group was higher than ANXA2 expression in Tis-T1 group and ANXA2 expression in patients who recurred at < 6 months after initial surgery was significantly higher than ANXA2 expression in patients who recurred at > 2 years (Hu et al., 2016). The role of ANXA2 expression in the acquisition of drug resistance is of interest, but there were few reports of ANXA2 expression in bladder cancer. P-glycoprotein interacted with ANXA2 and that both P-glycoprotein and ANXA2 expression were highly expressed in adriamycin-resistant breast cancer cell lines (Zhang et al., 2009). In another study, phosphorylation at tyrosine 23 is required for ANXA2 activation, but knockdown of P-glycoprotein using small interference RNA in adriamycin-resistant breast cancer cell lines was found to significantly inhibit tyrosine phosphorylation of ANXA2. P-glycoprotein may promote the invasion of MDR breast cancer cells by modulating the tyrosine phosphorylation of ANXA2 (Zhang et al., 2014). In the present study of bladder cancer, ANXA2 expression was significantly correlated with P-glycoprotein expression and was closely related with IVR. The interaction between ANXA2 and P-glycoprotein may be related to resistant mechanism against adriamycin in clinical samples of bladder cancer.

NPM is a nucleolar phosphoprotein and enhances cell division and growth presumably through stimulatory effects on ribosomal DNA transcription, ribosome subunit export and DNA replication during S-phase. In bladder cancer, higher NPM expression was shown to be linked to more advanced tumor stages, grades, likelihood of recurrence and poor prognosis (Tsui et al., 2008). In upper tract urothelial carcinoma, increased NPM expression is a strong predictor of extraurothelial recurrence after nephroureterectomy (Sawazaki et al., 2016) but NPM expression is not significantly associated with IVR in that study (unpublished data). From the current study in NMIBC, NPM expression did not appear to have significant relationship to intraluminal seeding which possibly caused IVR.

According to the EAU guideline, NMIBC patients were categorized into three groups (low, intermediate, high). In low-risk group, immediate single instillation of chemotherapy is recommended. In intermediate-risk group, adjuvant BCG instillation or chemotherapy instillation with/without immediate single instillation of chemotherapy is recommended. In high-risk group, adjuvant BCG instillation is recommended. In our study, the percentages of patients with high P-glycoprotein expression were 0% in low-risk, 33.3% in intermediate-risk, and 53.3% in high-risk group, respectively. High P-glycoprotein expression was a significant predictive factor for IVR after immediate single adriamycin instillation on univariate analysis. In the high-risk group, IVRFS rate of patients with high P-glycoprotein expression tended to be lower than that of patients with low P-glycoprotein expression. Although single adriamycin instillation is effective in low-risk group (the 2-year IVRFS rate: 85.7%), adjuvant BCG instillations should be considered in high-risk group to overcome resistance to adriamycin.

This study has several limitations. First, it was a non-randomized, retrospective, and single-center study on a limited number of patients. Second, intravesical adriamycin instillation was performed by two patterns (adriamycin 60 mg in 30 mL of normal saline or adriamycin 80 mg in 1,000 mL of normal saline). Nonetheless, there was not significant difference of IVR between patients with bolus adriamycin instillation and patients with continuous adriamycin irrigation. This study revealed statistical significance of P-glycoprotein in IVR in spite of the evaluation of a small number of patients. A prospective study with a large number of the patients is warranted to further clarify the prognostic role of P-glycoprotein expression. 

In Conclusion, p-glycoprotein expression was significantly associated with IVR in patients with NMIBC undergoing initial TURBT and immediate single postoperative intravesical adriamycin instillation. P-glycoprotein expression may provide an-important additional information about IVR. Further investigation is necessary to clarify the role of P-glycoprotein expression in bladder cancer for clinical use.

## Author Contribution Statement

All authors contributed to the study conception and design. Project development, data collection and analysis were performed by H. Sawazaki. Manuscript editing and data analysis were performed by K. Ito and H. Tsuda. The first draft of the manuscript was written by H. Sawazaki and all authors commented on previous versions of the manuscript. All authors read and approved the final manuscript.

## References

[B1] Abdallah HM, Al-Abd AM, El-Dine RS, El-Halawany A (2015). P-glycoprotein inhibitors of natural origin as potential tumor chemo-sensitizers: A review. J Adv Res.

[B2] Borst P, Evers R, Kool M, Wijnholds J (1999). The multidrug resistance protein family. Biochim Biophys Acta.

[B3] Bosschieter J, Nieuwenhuijzen JA, van Ginkel T (2018). Value of an immediate intravesical instillation of mitomycin C in patients with non-muscle invasive bladder cancer: a prospective multicenter randomized study in 2243 patients. Eur Urol.

[B4] Clifford SC, Thomas DJ, Neal DE, Lunec J (1994). Increased mdr1 gene transcript levels in high-grade carcinoma of the bladder determined by quantitative PCR-based assay. Br J Cancer.

[B5] Diestra JE, Condom E, Del Muro XG (2003). Expression of multidrug resistance proteins P-glycoprotein, multidrug resistance protein 1, breast cancer resistance protein and lung resistance related protein in locally advanced bladder cancer treated with neoadjuvant chemotherapy: biological and clinical implications. J Urol.

[B6] Hamada S, Horiguchi A, Asano T (2014). Prognostic impact of fatty acid synthase expression in upper urinary tract urothelial carcinoma. Jpn J Clin Oncol.

[B7] Hu H, Zhao J, Zang M (2016). Expression of Annexin A2 and its correlation with drug resistance and recurrence of bladder cancer. Technol Cancer Res Treat.

[B8] Inokuchi J, Narula N, Yee DS (2009). Annexin A2 positively contributes to the malignant phenotype and secretion of IL-6 in DU145 prostate cancer cells. Int J Cancer.

[B9] Kamat AM, Hahn NM, Efstathiou JA (2016). Bladder cancer. Lancet.

[B10] Lindstrom MS (2011). NPM/B23: a multifunctional chaperone in ribosome biogenesis and chromatin remodeling. Biochem Res Int.

[B11] Meng Q, Lei T, Zhang M (2013). Identification of proteins differentially expressed in Adriamycin-resistant (pumc-91/ADM) and parental (pumc-91) human bladder cancer cell lines by proteome analysis. J Cancer Res Clin Oncol.

[B12] Mitsui Y, Yasumoto H, Arichi N (2012). Current chemotherapeutic strategies against bladder cancer. Int Urol Nephrol.

[B13] Nargund VH, Tanabalan CK, Kabir MN (2012). Management of non-muscle-invasive (superficial) bladder cancer. Semin Oncol.

[B14] Obata K, Ohashi Y, Akaza H (1994). Prophylactic chemotherapy with intravesical instillation of Adriamycin and oral administration of 5-fluorouracil after surgery for superficial bladder cancer. The Japanese Urological Cancer Research Group for Adriamycin. Cancer Chemother Pharmacol.

[B15] Saginala K, Barsouk A, Aluru JS (2020). Epidemiology of Bladder cancer. Med Sci.

[B16] Sawazaki H, Ito K, Asano T (2016). Increased nucleophosmin expression is a strong predictor of recurrence and prognosis in patients with N0M0 upper tract urothelial carcinoma undergoing radical nephroureterectomy. World J Urol.

[B17] Tada Y, Wada M, Migita T (2002). Increased expression of multidrug resistance-associated proteins in bladder cancer during clinical course and drug resistance to doxorubicin. Int J Cancer.

[B18] Tsui KH, Juang HH, Lee TH (2008). Association of nucleophosmin/B23 with bladder cancer recurrence based on immunohistochemical assessment in clinical samples. Acta Pharmacol Sin.

[B19] Xu XH, Pan W, Kang LH, Feng H, Song YQ (2015). Association of annexin A2 with cancer development (Review). Oncol Rep.

[B20] Zamboni S, Baumeister P, Mattei A (2019). Single postoperative instillation for non-muscle invasive bladder cancer: are there still any indication?. Transl Androl Urol.

[B21] Zhang F, Zhang L, Zhang B (2009). Anxa2 plays a critical role in enhanced invasiveness of the multidrug resistant human breast cancer cells. J Proteome Res.

[B22] Zhang F, Zhang H, Wang Z (2014). P-glycoprotein associates with Anxa2 and promotes invasion in multidrug resistant breast cancer cells. Biochem Pharmacol.

